# A Novel Mathematical Approach to Define the Genes/SNPs Conferring Risk or Protection in Sporadic Amyotrophic Lateral Sclerosis Based on Auto Contractive Map Neural Networks and Graph Theory

**DOI:** 10.1155/2012/478560

**Published:** 2012-08-09

**Authors:** Massimo Buscema, Silvana Penco, Enzo Grossi

**Affiliations:** ^1^Semeion Research Center, Via Sersale 117, 00128 Rome, Italy; ^2^Medical Genetics, Department of Laboratory Medicine, Niguarda Ca' Granda Hospital Piazzaza Ospedale Maggiore 3, 20100 Milan, Italy; ^3^Medical Department, Bracco SpA, Via E. Folli 50, 20134 Milan, Italy

## Abstract

*Background*. Complex diseases like amyotrophic lateral sclerosis (ALS) implicate phenotypic and genetic heterogeneity. Therefore, multiple genetic traits may show differential association with the disease. The Auto Contractive Map (AutoCM), belonging to the Artificial Neural Network (ANN) architecture, “spatializes” the correlation among variables by constructing a suitable embedding space where a visually transparent and cognitively natural notion such as “closeness” among variables reflects accurately their associations. *Results*. In this pilot case-control study single nucleotide polymorphism (SNP) in several genes has been evaluated with a novel data mining approach based on an AutoCM. We have divided the ALS dataset into two dataset: Cases and Control dataset; we have applied to each one, independently, the AutoCM algorithm. Six genetic variants were identified which differently contributed to the complexity of the system: three of the above genes/SNPs represent protective factors, APOA4, NOS3, and LPL, since their contribution to the whole complexity resulted to be as high as 0.17. On the other hand ADRB3, LIPC, and MMP3, whose hub relevancies contribution resulted to be as high as 0.13, seem to represent susceptibility factors. *Conclusion*. The biological information available on these six polymorphisms is consistent with possible pathogenetic pathways related to ALS.

## 1. Background

Investigating the pattern of correlations among large numbers of variables in large databases is certainly a quite difficult task that is seriously demanding in both computational time and capacity. The statistically oriented literature has developed a variety of methods with different power and usability, all of which, however, share a few basic problems, among which the most outstanding are the nature of the a priori assumptions that have to be made on the data-generating process, the near impossibility to compute all the joint probabilities among the vast number of possible couples and n-tuples that are in principle necessary to reconstruct the underlying process' probability law, and the difficulty of organizing the output in an easily grasped, ready-to-access format for the nontechnical analyst. The consequence of the first two weaknesses is the fact that when analyzing poorly understood problems characterized by heterogeneous sets of potentially relevant variables, traditional methods can become very unreliable when not unusable. The consequence of the last one is that, also in the cases where traditional methods manage to provide a sensible output, their statement and implications can be so articulated to become practically unuseful or, even worse, easily misunderstood. 

In this paper, we introduce a new methodology based on an Artificial Neural Network (ANN) architecture, the Auto Contractive Map (AutoCM) [1], which allows for basic improvements in both robustness of use in badly specified and/or computationally demanding problems and output usability and intelligibility. In particular, AutoCMs “spatialize” the correlation among variables by constructing a suitable embedding space where a visually transparent and cognitively natural notion such as “closeness” among variables reflects accurately their associations. Through suitable optimization techniques that will be introduced and discussed in detail in what follows, “closeness” can be converted into a compelling graph-theoretic representation that picks all and only the relevant correlations and organizes them into a coherent picture. Such representation is not actually constructed through some form of cumbersome aggregation of two-by-two associations between couples of variables but rather by building a complex global picture of the whole pattern of variation. Moreover, it fully exploits the topological meaningfulness of graph-theoretic representations in that actual paths connecting nodes (variables) in the representation carry a definite meaning in terms of logical interdependence in explaining the data set's variability. We are aware of the fact that these techniques are novel and therefore not entirely understood so far in all of their properties and implications. However, we are convinced that their actual performance in the context of well-defined, well-understood problems provides an encouraging test to proceed in this direction. We applied this new approach in Amyotrophic Lateral Sclerosis (ALS), a fatal neurodegenerative condition causing progressive motor neuron loss, leading to death within a few years of onset. There is no effective treatment for this devastating disease, although riluzole is reported to have a mild effect in slowing its progression [2, 3]. This failure is likely to be related to the poor knowledge of the pathogenetic mechanisms of ALS, as well as to its heterogeneity. Genetic factors are known to play an important role; at least nine of genetically transmitted forms of ALS are known and several genes possibly influencing the occurrence and the phenotypical expression of ALS have been identified [4, 5]. However, the knowledge of the genes that may play a role and of the mechanisms by which they may cause the phenotype is still very incomplete. The identification of genes that underlie the sporadic and the genetic forms of ALS will be highly relevant since it will identify novel metabolic pathways involved in neurodegeneration.

Previously we approached the genetic of sporadic ALS (SALS) disease with artificial neural networks to identify a possible genetic background predisposing to the sporadic form. A dataset containing genetic data from 54 SALS cases and 208 controls was analyzed with three different analytical approaches: Linear Discriminant Analysis, Standard Artificial Neural Networks, and Advanced Intelligent Systems; with this latter approach the predictive accuracy to discriminate between cases and controls reached an average of 96% (range 94.4 to 97.6). In addition we identified seven genetic variants essential to differentiate cases from controls [6].

The obtained results point out the need to employ systems really able to handle the disease complexity instead of treating the data with reductionistic approaches unable to detect multiple genes of smaller effect predisposing to the disease.

We report here the application of a new developed analytical approach to the SALS dataset, based on Auto-CM system and Maximally regular graph theory.

The idea was to test the power of this new algorithm in a medical context such as SALS disease to shed light on the puzzling of the disease.

## 2. Methods

### 2.1. Database

We used a previously described dataset [6]. Briefly, genotypes derived from 60 biallelic polymorphisms within 35 genes that were selected from pathways of lipid and homocysteine metabolism, regulation of blood pressure, coagulation, inflammation, cellular adhesion, and matrix integrity. All subjects tested were of Caucasian origins, 54 were SALS and 208 were controls. SALS patients consist of 28 males (56.4 years; 46.9–65.8) and 26 females (62.9 years; 57.8–67.9), mean age at onset of disease 59.62 years; (range 53.7–65.5 years), clinical onset was spinal in 61.1% (33/54) and bulbar in 38.9% (21/54) of cases, mean disease duration at the time of observation was 3.2 years ( range 1–10 years).

Controls subjects were 144 males and 67 females; age range from 21 to 75 years, (average 38.94).

### 2.2. The Auto Contractive Map

We begin our analysis with a relatively concise but technically detailed presentation of the ANN architecture that provides the basis for all of the subsequent analysis: the Auto Contractive Map (AutoCM) [7, 8]. The AutoCM is characterized by a three-layer architecture: an input layer, where the signal is captured from the environment, a hidden layer, where the signal is modulated inside the AutoCM, and an output layer, through which the AutoCM feeds back upon the environment on the basis of the stimuli previously received and processed.

**Figure 1:**
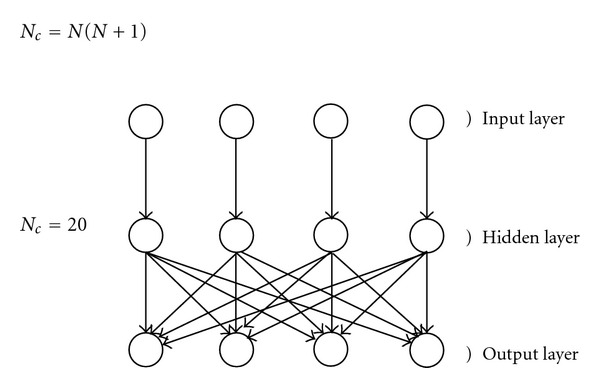
An example of an AutoCM with *N* = 4.

Each layer contains an equal number of *N* units, so that the whole AutoCM is made of 3*N* units. The connections between the Input and the Hidden layers are monodedicated, whereas the ones between the hidden and the output layers are fully saturated, that is, at maximum gradient. Therefore, given *N* units, the total number of the connections, N_c_, is given by Figure 1.

All of the connections of AutoCM may be initialized either by assigning a same, constant value to each, or by assigning values at random. The best practice is to initialize all the connections with a same, positive value, close to zero.

The learning algorithm of AutoCM may be summarized in a sequence of four characteristic steps:

1. signal transfer from the input into the hidden layer;
2. adaptation of the values of the connections between the input and the hidden layers;
3. signal transfer from the hidden into the output layer;
4. adaptation of the value of the connections between the hidden and the output layers.

Notice that steps 2 and 3 may take place in parallel.

We write as *m* ^[*s*]^ the units of the input layer (sensors), scaled between 0 and 1; as *m* ^[*h*]^ the units of the hidden layer as *m* ^[*t*]^ the units of the output layer (system target). We moreover define **v**, the vector of monodedicated connections; **w**, the matrix of the connections between the hidden and the output layers; *n*, the discrete time that spans the evolution of the AutoCM weights, or, put another way, the number of cycles of processing, counting from zero and stepping up one unit at each completed round of computation: *n* ∈ *T*.

In order to specify the steps 1–4 that define the AutoCM algorithm, we have to define the corresponding signal forward-transfer equations and the learning equations, as follows.

(a) Signal transfer from the input to the hidden layer:
  (1) $$\begin{array}{c} m_{i_{\left(n\right)}}^{\left[h\right]}=m_{i}^{\left[s\right]}\left(1-\frac{v_{i_{\left(n\right)}}}{C}\right), \end{array}$$
  where *C* is a positive real number not lower than 1, which we will refer to as the contraction parameter (see below for comments), and where the (*n*) subscript has been omitted from the notation of the input layer units, as these remain constant at every cycle of processing. It is useful to set $C=\sqrt[{2}]{N}$, where *N* is the number of variables considered.
(b) Adaptation of the connections *v*_*i*_(*n*)__through the variation Δ*v*_*i*_(*n*)__, which amounts to trapping the energy difference generated according to (1):
  (2) $$\begin{array}{c} \Delta v_{i_{\left(n\right)}}=\left(m_{i}^{\left[s\right]}-m_{i_{\left(n\right)}}^{\left[h\right]}\right)\cdot \left(1-\frac{v_{i_{\left(n\right)}}}{C}\right),\\ v_{i_{\left(n+1\right)}}=v_{i_{\left(n\right)}}+\alpha \cdot \Delta v_{i_{\left(n\right)}}. \end{array}$$
(c) Signal transfer from the hidden to the output layer:
  (3) $$\begin{array}{c} \text{Ne}{\text{t}}_{i_{\left(n\right)}}=\sum_{j=1}^{N}m_{j_{\left(n\right)}}^{\left[h\right]}\cdot \left(1-\frac{w_{i,j_{\left(n\right)}}}{C}\right), \end{array}$$
  (4) $$\begin{array}{c} m_{i_{\left(n\right)}}^{\left[t\right]}=m_{i_{\left(n\right)}}^{\left[h\right]}\left(1-\frac{\text{Ne}{\text{t}}_{i_{\left(n\right)}}}{C}\right). \end{array}$$
(d) Adaptation of the connections *w*_*i*,*j*_(*n*)__through the variation Δ*w*_*i*,*j*_(*n*)__, which amounts, accordingly, to trapping the energy difference as to (4):
  (5) $$\begin{array}{c} \Delta w_{i,j_{\left(n\right)}}=\left(m_{i_{\left(n\right)}}^{\left[h\right]}-m_{i_{\left(n\right)}}^{\left[t\right]}\right)\cdot \left(1-\frac{w_{i,j_{\left(n\right)}}}{C}\right)\cdot m_{j_{\left(n\right)}}^{\left[h\right]},\\ w_{i,j_{\left(n+1\right)}}=w_{i,j_{\left(n\right)}}+\alpha \cdot \Delta w_{i,j_{\left(n\right)}}. \end{array}$$

First of all, we need to specify that *α* is the learning coefficient of AutoCM. This coefficient has to be chosen taking into consideration 3 different condition:

1. AutoCM weights are updated at every cycle;
2. the order of selection of any record at each epoch is random (a epoch is the number of cycles we need to update every record of the dataset);
3. after every cycle the AutoCM is closer to its converge point, *T*, and the amount of updating between *n* = 0 and *n* = *T* decreases up to zero.

For this reason it is necessary to set up the learning coefficient in a way that AutoCM can update its weights after a reasonable number of epochs, without to be influenced by the random order of the records at each cycle.

Consequently, we suggest to chose the learning coefficient taking into account the contractive factor, *C*, the number of variables, *N*, and the number of records, *M*, of the assigned dataset:

(6) $$\begin{array}{c} \alpha =\frac{N}{M\cdot C}. \end{array}$$

### 2.3. AutoCMs: A Theoretical Discussion

There are a few important peculiarities of Auto-CMs [9–12] with respect to more familiar classes of ANNs that need special attention and call for careful reflection.

(i) AutoCMs are able to learn also when starting from initializations where all connections are set at the same value, that is, they do not suffer the problem of the symmetric connections.
(ii) During the training process, AutoCMs always assign positive values to connections. In other words, Auto-CMs do not allow for inhibitory relations among nodes, but only for different strengths of excitatory connections.
(iii) AutoCMs can learn also in difficult conditions, namely, when the connections of the main diagonal of the second layer connection matrix are removed. In the context of this kind of learning process, Auto-CMs seem to reconstruct the relationship occurring between each couple of variables. Consequently, from an experimental point of view, it seems that the ranking of its connections matrix translates into the ranking of the joint probability of occurrence of each couple of variables.
(iv) Once the learning process has occurred, any input vector, belonging to the training set, will generate a null output vector. So, the energy minimization of the training vectors is represented by a function trough which the trained connections absorb completely the input training vectors. Thus, AutoCM seems to learn how to transform itself in a “dark body”.
(v) At the end of the training phase (Δ*w*_*i*,*j*_ = 0), all the components of the weights vector **v**reach up the same value:
  (7) $$\begin{array}{c} \underset{n\to \infty }{\lim}v_{i_{\left(n\right)}}=C. \end{array}$$
  The matrix **w**, then, represents the AutoCM knowledge about the whole dataset.

One can use the information embedded in the **w** matrix to compute in a natural way the joint probability of occurrence among variables:

(8) $$\begin{array}{c} p_{i,j}=\frac{w_{i,j}}{\sum_{j=1}^{N}w_{i,j}}; \end{array}$$

(9) $$\begin{array}{c} P\left(m_{j}^{\left[s\right]}\right)=\sum_{i}^{N}p_{i,j}=1. \end{array}$$

The new matrix **p** can be read as the probability of transition from any state variable to anyone else:

(10) $$\begin{array}{c} P\left(m_{i}^{\left[t\right]}|m_{j}^{\left[s\right]}\right)=p_{i,j}. \end{array}$$

(i) Alternatively, the matrix **w** may be transformed into a non-Euclidean distance metric (semimetric), when we train the AutoCM with the main diagonal of the **w** matrix fixed at value *N*. Now, if we consider *N* as a limit value for all the weights of the **w** matrix, we can write
  (11) $$\begin{array}{c} d_{i,j}=N-w_{i,j}. \end{array}$$
  The new matrix **d** is again a squared symmetric matrix, where the main diagonal entries are null (i.e., they represent the zero distance of each variable from itself), and where the off-diagonal entries represent “distances” between each couple of variables.

### 2.4. AutoCM and Minimum Spanning Tree

Equation (11) transforms the squared weights matrix of AutoCM into a squared matrix of distances among nodes. Each distance between a pair of nodes may therefore be regarded as the weighted edge between these pair of nodes in a suitable graph-theoretic representation, so that the matrix **d** itself may be analyzed through the graph theory toolbox.

A graph is a mathematical abstraction that is useful for solving many kinds of problems. Fundamentally, a graph consists of a set of vertices, and a set of edges, where an edge is an object that connects two vertices in the graph. More precisely, a graph is a pair (*V*,* E*), where *V* is a finite set and *E* is a binary relation on *V*, to which it is possible to associate scalar values (in this case, the distances *d*_*i*,*j*_). 

At this point, it is useful to introduce the concept of Minimum Spanning Tree (M.S.T.) [9–12].

The Minimum Spanning Tree problem is defined as follows: find an acyclic subset *T* of *E* that connects all of the vertices in the graph and whose total weight (namely, the total distance) is minimized, where the total weight is given by:

(12) $$\begin{array}{c} d\left(T\right)=\sum_{i=0}^{N-1}\sum_{j=i+1}^{N}d_{i,j},\;\forall d_{i,j}. \end{array}$$

*T*  is called a spanning tree, and the MST is the *T* whose weighted sum of edges attains the minimum value:

(13) $$\begin{array}{c} \text{Mst}=\mathrm{Min}\left\{d\left(T_{k}\right)\right\}. \end{array}$$

From conceptual point of view, the MST represents the energy minimization state of a structure. In fact, if we consider the atomic elements of a structure as vertices of a graph and the strength among them as the weight of each edge, linking a pair of vertices, the MST represents the minimum of energy needed so that all the elements of the structure preserve their mutual coherence. In a closed system, all the components tend to minimize the overall energy. So the MST, in specific situations, can represent the most probable state for the system to tend.

To determine the MST of an undirected graph, each edge of the graph has to be weighted. Equation (11) shows a way to weight each edge whose nodes are the variables of a dataset, and where the weights of a trained AutoCM provide the (weight) metrics.

Obviously, it is possible to use any kind of Auto-Associative ANN or any kind of Linear Auto-Associator to generate a weight matrix among the variables of an assigned dataset. But it is hard to train a two-layer Auto-Associative Back Propagation ANN with the main diagonal weights fixed (to avoid autocorrelation problems). In most cases, the Root Mean Square Error (RMSE) stops to decrease after a few epochs, and especially when the orthogonality of the records is relatively high, a circumstance that is frequent when it is necessary to weight the distance among the records of the assigned dataset. In this case, it is necessary to train the transposed matrix of the dataset. By the way, if a linear Auto-Associator is used to the purpose, all of the nonlinear association among variables will be lost.

Therefore, AutoCM seems to be the best choice to date to compute a complete and a nonlinear matrix of weights among variables or among records of any assigned dataset.

### 2.5. Graph Complexity: The *H* Function

Now we introduce a new indicator: the degree of protection of each node in any a directed graph.

This indicator defines the rank of centrality of each node within the graph, when an iterative pruning algorithm is applied. The pruning algorithm was found and applied for the first time as a global indicator for graph complexity by Giulia Massini at Semeion Research Center in 2006 [13]: (See Algorithm 1).

The higher the rank of a node, the bigger the centrality of its position within the graph. The latest nodes to be pruned are also the kernel nodes of the graph. In the present paper, this algorithm is generalized to measure the global complexity of any kind of graph. 

The pruning algorithm can be used also to define the quantity of graph complexity of any graph. If we take *μ* as the mean number of nodes without any link, at each iteration, as the pruning algorithm is running, we can define the hubness Index, *H*_0_, of a graph with *N* nodes. In order to properly define this quantity, we need to introduce a few preliminary concepts. We define a cycle or iteration of the pruning algorithm as a given round of application of the algorithm. At each cycle, there corresponds a gradient, which can be different from cycle to cycle. Insofar as two subsequent cycles yield the same gradient, they belong to the same pruning class. As the gradient changes degli one cycle to the other, the previous class ends and a new one begins. We are now in the position to define hubness as follows:

(14) $$\begin{array}{c} H_{0}=\frac{\mu \cdot \varphi -1}{A};\;0<H_{0}<2; \end{array}$$

(15) $$\begin{array}{c} \mu =\frac{1}{M}\sum_{i}^{M}Nd_{i}=\frac{N}{M}; \end{array}$$

(16) $$\begin{array}{c} \varphi =\frac{1}{P}\sum_{j}^{P}S_{\text{T}{\text{G}}_{j}}. \end{array}$$

*A* is number of links of the graph (*N-1* for trees); *N* is Number of Nodes; *M* is number of cycles of the pruning algorithm; *P* is number of states implied into a change of gradient, during the pruning process; *Nd*_*i*_ is number of nodes without link at the *j*-th iteration; *S*_TG_*j*__ is summation of the gradient of the states implied into a change of gradient, during the pruning process.

Equation (15) measures the mean gradient of the graph.

Equation (16) measures the dynamics of the gradient changes during the pruning process.

Equation (14) is a complex ratio between the mean gradient and the dynamics of this gradient, from one side and the global graph connectivity from the other side.

Using *H*_0_ as a global indicator, it is possible to define to what extent a graph is hub oriented.

The *H* indicator (14), (15), and (16) represents the global hubness of graph. When *H* = 0, the tree is a one-dimensional line and its complexity is minimal. When *H* = 1, the tree presents only one hub, and its complexity is the maximum than a tree can attain. The complexity of a graph, in fact, is connected to its entropy. The quantity of information in a graph is linked to the graph diameter and to the connectivity of the vertices: given the number of vertices, the shorter the diameter, the bigger the entropy. Starting from the classical notion of entropy we can thus write

(17) $$\begin{array}{c} E=-K\cdot \sum_{i}^{N}p_{i}\cdot \ln\left(p_{i}\right). \end{array}$$

If we name *E*(*G*) the topological entropy of a generic tree-graph, we can write

(18) $$\begin{array}{c} E\left(G\right)=-\frac{A}{M}\cdot \sum_{i}^{N}\frac{C_{i}}{A}\cdot \ln\left(\frac{C_{i}}{A}\right);\;0<E\left(G\right)<\infty , \end{array}$$

Where *A* is number of graph edges (*N* − 1, when the graph is a tree); *N* is number of graph vertices; *M* is number of pruning cycles necessary to deconnect the graph completely; *C*_*i*_ is degree of connectivity of each vertex.

The quantity *C*_*i*_/*A* measures the probability that a generic node *C*_*j*_, where *j* ≠ *i*, has to be directly linked to the node *C*_*i*_. This means that the entropy of a graph, *E*(*G*), will increase when the number of vertices with a large number of links increases. Accordingly, the probability to arrange the links of *N* vertices, using a random process, into a linear chain is the lowest. Therefore, the higher the number of pruning cycles, *M*, needed for a graph, the smaller is graph entropy. Equation (18) shows clearly that a “hub tree” has more entropy than a “chain tree”. Consequently, when the *H* index of a tree increases, its redundancy increases as well.

### 2.6. The Delta *H* Function

Considering how the structure of a given graph is changed by a pruning process, it becomes natural to think of what happens to graphs, and in particular to MSTs, as one or more of their nodes are deleted. In which way will the graph has to be organized to continue to reflect as best as possible the underlying structure of relationships once one or more nodes are taken away? How will the other nodes rearrange their links on the basis of the underlying metric and constraints, to connect each other once again?

Define a *H* index for each one of *N* different MSTs, generated from the original distance matrix by deleting one different vertex at each step:

(19) $$\begin{array}{c} H_{i}=\frac{\mu_{i}\cdot \varphi_{i}-1}{A-1};\;\;0<H_{i}<2;\\ \mu_{i}=\frac{1}{M}\sum_{j}^{M}Nd_{j}=\frac{N}{M};\\ \varphi_{i}=\frac{1}{P}\sum_{k}^{P}S_{TG_{k}\;}. \end{array}$$

*A* is number of links of the graph (*N* − 1 for trees); *N* is Number of Nodes; *M* is number of cycles of the pruning algorithm; *P* is number of states implied into a change of gradient, during the pruning process; *Nd*_*i*_ is number of nodes without link at the *j*-the iteration; *S*_*TG*_*k*__ is Summation of the gradient of the states implied into a change of gradient, during the pruning process.

Each *H*_*i*_ represents the tree complexity of the same, original distance matrix when the *i* ^th^ vertex is deleted. Consequently, the difference between the complexity of the whole MST (i.e., *H*_0_) and the complexity of any of the MSTs that are obtained by deleting one of the graph vertices (*H*_*i*_), is the measure of the contribution of that specific (*i*) vertex of the graph to the original graph's global complexity:

(20) $$\begin{array}{c} \delta H_{i}=H_{0}-H_{i}. \end{array}$$

This new index measures to what extent each vertex of a graph contributes to increase (*δH*_*i*_ < 0) or to decrease (*δH*_*i*_ > 0) the redundancy of the original, overall graph. We have named this function Delta *H* function; it can be applied to any kind of graph.

### 2.7. AutoCM and Maximally Regular Graph (MRG)

The MST represents what we could call the “nervous system” of any dataset. In fact, summing up all of the connection strengths among all the variables, we get the total energy of that system. The MST selects only the connections that minimize this energy, that is, the only ones that are really necessary to keep the system coherent. Consequently, all the links included in the MST are fundamental, but, on the contrary, not every “fundamental” link of the dataset needs to be in the MST. Such limit is intrinsic to the nature of MST itself: every link that gives rise to a cycle into the graph (viz., that destroys the graph's “treeness”) is eliminated, whatever its strength and meaningfulness. To fix this shortcoming and to better capture the intrinsic complexity of a dataset, it is necessary to add more links to the MST, according to two criteria:

- the new links have to be relevant from a quantitative point of view;
- the new links have to be able to generate new cyclic regular microstructures, from a qualitative point of view.

Consequently, the MST tree-graph is transformed into an undirected graph with cycles. Because of the cycles, the new graph is a dynamic system, involving in its structure the time dimension. This is the reason why this new graph should provide information not only about the structure but also about the functions of the variables of the dataset. 

To build the new graph, we need to proceed as follows:

- assume the MST structure as the starting point of the new graph;
- consider the sorted list of the connections skipped during the derivation of the MST;
- estimate the *H* function of the new graph each time that you add a new connection to the MST basic structure, to monitor the variation of the complexity of the new graph at every step.

We will call Maximally Regular Graph (MRG) the graph whose H function attains the highest value among all the graphs generated by adding back to the original MST, one by one, the missing connections previously skipped during the computation of the MST itself. Starting from (14), the MRG may be characterized as follows:

(21) Hi=f(G(Ai,N))/“Generic Function on a graph with  Ai  arcs and  N  nodes at  i  th test” Hi=μi·φi−  1Ai/“Calculation of  H  Function, where  H0  represents MST complexity”/H∗=Max⁡{Hi}/“Graph with highest  H= MRG”/R∗=Max⁡ arg{Hi}/“Number of links added by MRG”/i∈[0,1,2,…,R]/“Index of  H  Function”/N−1<Ai<N·(N−1)2“interval of the number of graph arcs”/R∈[0,1,…,(N−1)·(N−2)2]/“Number of the skipped arcs during the MST generation”/

The *R* number is a key variable during the computation of the MRG. *R* could in fact be also null, when the computation of the MST calls for no connections to be skipped. In this case, there is no MRG for that dataset.

*R*, moreover, makes sure that the last, and consequently the weakest, connection added to generate the MRG is always more relevant that the weakest connection of the MST. The MRG, finally, generates, starting from the MST, the graph presenting the highest number of regular microstructures that make use of the most important connections of the dataset. The higher the value of the *H* Function at the connections selected to generate the MRG, the more meaningful the microstructures of the MRG. 

The MRG calculation is also useful to define the MST compactness: less is the number of arcs skipped during the MST generation, more the MST is representative; in other terms:

(22) $$\begin{array}{c} \text{compactness}\;\;\left(\text{Mst}\right)\\ \phi =1.0-\frac{R}{P};\\ P=\frac{\left(N-1\right)\cdot \left(N-2\right)}{2}. \end{array}$$

Another important index is the Relevance of MRG: this index depends on 2 other indexes:

(i) the MRG Hubness, H*, (21):
  (23) $$\begin{array}{c} \overset{•}{H}=\frac{H^{*}-H_{0}}{H^{*}} \end{array}$$
(ii) and the number of new links added by MRG generation:
  (24) $$\begin{array}{c} \overset{•}{R}=\frac{R^{*}}{R}. \end{array}$$

The fuzzy combination of these two indexes can express the MRG Relevance:

(25) $$\begin{array}{c} \text{relevance}\;\;\left(\text{MRG}\right)\\ \varphi =\left(\overset{•}{H}+\overset{•}{R}\right)-\left(\overset{•}{H}\cdot \overset{•}{R}\right). \end{array}$$

At this point it is we can approximate a new index to measure the amount of information provided by MRG respect to MST:

(26) $$\begin{array}{c} \text{information}\;\;\left(\text{MRG}\right)\\ \psi =\phi \cdot \varphi . \end{array}$$

## 3. Results

**Figure 2:**
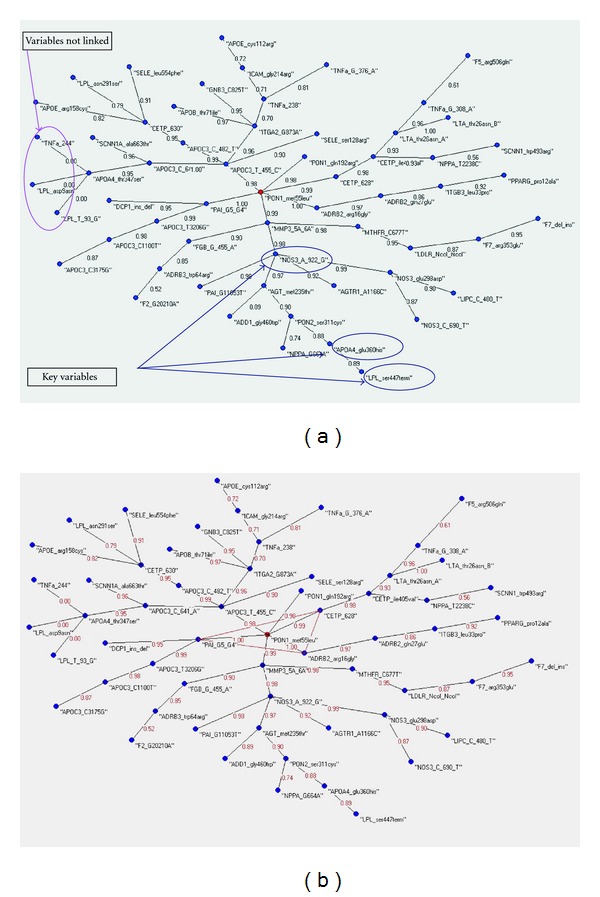
(a) The MST of the cases databest. Into the blue circles the key variables of the graph. (b) The MST of the controls databest. Into the red circles the key variables of the graph.

We have divided the ALS dataset into: the Cases dataset (58 records) and the Control dataset (207 records). Then we have independently applied to each one the AutoCM algorithm. The AutoCM algorithm generates two weighted MST and the Delta H function points out the key variables of the two datasets (see Figure 2(a) and Figure 2(b)). The two MSTs are different topologically and locally (different variables connections) and the Delta H function shows a very interesting situation (see Table 1).

- 3 variables (APOA4_glu360his, NOS3_A_922_G, LPL_ser447term) seem to be the reason of the low complexity of the cases MST: when each one of them is removed, the MST increases its complexity, taking the same *H* value of the global MST of the control dataset (*H* Cases = 0.171429 versus *H* Control = 0.17193);
- 3 variables (ADRB3_trp64arg, LIPC_C_480_T, MMP3_5A_6A) seem to be the reason of the high complexity of the control MST: when each one of them is removed, the MST decreases its complexity, taking the same *H* value of the global MST of the cases dataset (*H* Cases = 0.137427 versus *H* Control = 0.136905);

If these considerations should have a biological reason, the AutoCM algorithm and the Delta Function procedure have shown to be very capable to catch the hidden information into the medical datasets.

**Figure 3:**
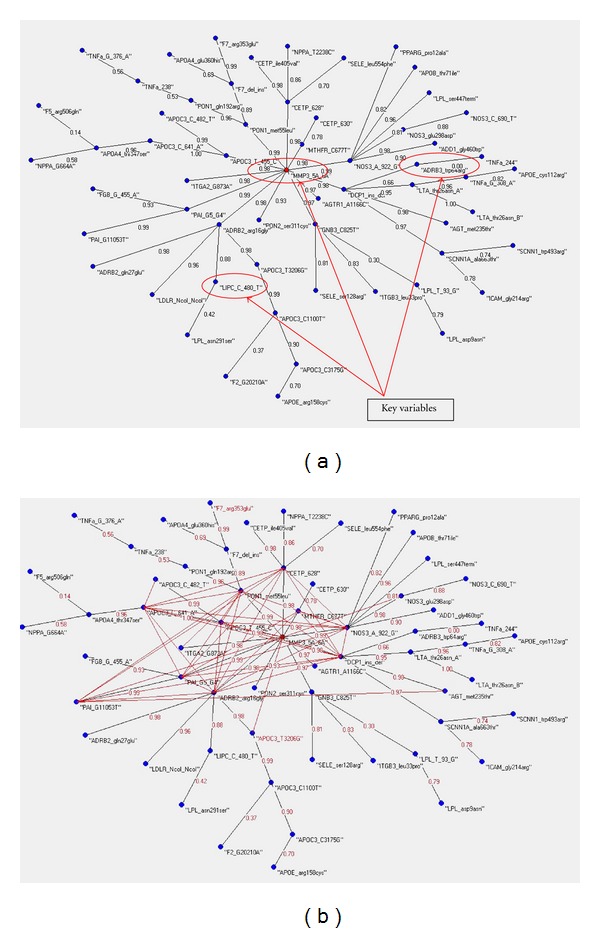
(a) The MRG of the cases databest. In red the MRG connections. (b) The MRG of the controls databest. In red the MRG connections.

As a second step of this analysis, we have calculated the MRG of the two dataset (see Figures 3(a) and 3(b)). Also in this case the MRG shows a low complexity of hubbness and Links in the cases dataset and a very high complexity in the control dataset. This seems to confirm that in an ideal health condition the living organisms manifest a high ratio of complex regularity and redundancy of structures and functions.

## 4. Discussion

Healthy physiologic function is characterized by a complex interaction of multiple control mechanisms that enable an individual to adapt to the exigencies and unpredictable changes of everyday life. The disease process appears to be marked by a progressive impairment in these mechanisms, resulting in a loss of dynamic range in physiologic function and, consequently, a reduced capacity to adapt to stress. The emerging concept is that loss of redundancy, entropy and complexity is an hallmark of disease and in particular of chronic diseases.

Defining and quantifying the complexity of variables interactions are very difficult tasks from a mathematical point of view. Complex network theory by establishing criteria to define hubs in a particular variables network provides a framework on which building up parameters corresponding to an increase or loss of complexity in relation to the presence or absence of a particular variable in a variables set.

In this paper we have applied a novel revolutionary methodology to establish which of polymorphisms potentially involved in SALS occurrence play a fundamental role in protecting or in increasing the vulnerability for the disease occurrence increasing or reducing the hubness of a graph encoding the dynamic relation among genotypes many to many.

Six genetic variants were identified which differently contributed to the complexity of the system: apolipoprotein A-IV (APOA-IV) glu360his (rs5110), nitric oxide synthase 3 (NOS3)-922A/G (rs1800779), lipoprotein lipase (LPL) ser447term (rs328), adrenergic, beta-3 receptor (ADRB3) trp64arg (rs4994), hepatic lipase (LIPC)-480C/T (rs1800588) and matrix metallopeptidase 3 (MMP3)-1171 5A/6A (rs3025058). Three of the above genes/SNPs represent protective factors, APOA4 glu360his, NOS3-922A/G and LPL ser447term, since their contribution to the whole complexity resulted to be as high as 0.17 (see table 1). On the other hand ADRB3 trp64arg, LIPC-480C/T, and MMP3-1171 5A/6A, whose hub relevancies resulted to be as high as 0.13, seem to represent susceptibility factors (see Table 1).

Among the genes/SNPs conferring risk or protection from the disease, we noted that four of these are involved in the lipid pathways, APOA4, LPL, LIPC, ADRB3 while two are involved also in oxidative stress, angiogenesis, and cellular cytoskeletal (NOS3 and MMP3). 

The protective genes/SNPs here identified include the gene for apo A-IV, mapping on chromosome 11q2 and coding a glycoprotein whose primary translation product is a 396-residue preprotein which after proteolytic processing is secreted. Although its precise function is not known, apo A-IV is a potent activator of lecithin-cholesterol acyltransferase in vitro and displays antioxidant and antiatherogenic properties in vitro, and the antiatherogenic properties of apoA-IV suggest that this protein may act as an anti-inflammatory agent [14]. The second protective gene/SNPs still involved in lipid pathways is LPL ser447term; the gene maps on chromosome 8p22 and encodes a lipoprotein lipase, which is expressed in heart, muscle, and adipose tissue. LPL has the dual functions of triglyceride hydrolase and ligand/bridging factor for receptor-mediated lipoprotein uptake. Several DNA variants at the LPL gene locus have been found to be associated with the plasma lipid levels, in particular the Ser447ter has the potential to elevate the plasma high-density lipoprotein (HDL) levels [15]. The role of HDL in ALS disease is still controversial, hyperlipidemia was shown to be a significant prognostic factor for survival of patients with ALS, linked to a better outcome [16]. However, recent findings in Italian ALS patients did not support this observation, even though some evidence emerged that respiratory impairment, but not a worse clinical status or a lower body mass index, was related to a decrease in blood lipids and LDL/HDL ratio [17].

The last protective factor, NOS3-922A/G variant, belongs to a gene localized to chromosome 7q36 and coding the cytosolic enzyme of endothelial cells, a key actor in the process of modulation of vascular tone by producing nitric oxide (NO), a vasodilator agent. Constitutive NO release from microvascular endothelium seems to be responsible to prevent leukocyte margination under physiological conditions by modulating oxidative metabolism in endothelial cells. In this mechanism NO act as antioxidant agent to prevent the formation of iron-mediated hydroperoxide. Accumulating evidences indicate that ALS is associated with oxidative damage induced by free radicals. Enhancement of oxidative damage markers and signs of increased compensatory response to oxidative stress was found in patients with SALS [18], and since different antioxidant systems seem to be involved in ALS compared to other neurodegenerative diseases, oxidative stress may be a cause rather than a consequence of the neuronal death [19].

Considering now the vulnerability factors, the LIPC-480C/T belongs to a gene located on chromosome 15q21–23 and coding a glycoprotein involved in metabolism of several lipoproteins. The C/T substitution at −480 of the promoter region of the gene has been shown to be significantly associated to lower lipase activity [20] and it is also involved in anti-inflammatory and antioxidant activity [21]. Again, the lipid pathway is still involved.

The ADRB3 gene has been localized to chromosome 8p12-8p11.1 and it codes for a member of the adrenergic receptor group of G-protein-coupled receptors; it is located mainly in adipose tissue and is involved in the regulation of lipolysis and thermogenesis. Some *β*3 agonists have demonstrated antistress effects in animal studies, suggesting it also has a role in the CNS [22]. In addition, the trp64arg polymorphism seems to be associated to an increased BMI [23] and recently this polymorphism seems to be associated with elite endurance performance [24]. This is quite interesting since the literature supported the concept of soccer, and consequent head trauma, and ALS being interrelated, with high levels of athleticism/physical activity perhaps playing an additive part in the pathogenesis of the disease [25]. Even though the role of exposure to physical exercise, together with trauma, in ALS has been debated, in a recent pilot study comprising 61 patients and 112 controls the authors demonstrate that physical exercise but not with traumatic events [26] is related.

Regarding the last at risk factor, MMP3-1171 5A/6, this belongs to a gene mapping on chromosome 11q22.3 and coding a protein of the matrix metalloproteinase family (MMPs). MMPs a family of zinc-dependent endoproteinases, are effector molecules in the breakdown of the blood-brain and blood-nerve barrier, and promote neural tissue invasion by leukocytes in inflammatory diseases of the central and peripheral nervous systems. Moreover, MMPs play an important role in synaptic remodeling, neuronal regeneration, and remyelination [27]. MMPs have been suggested to play an important role in ALS pathology and several studies are still ongoing both in animal models as well as in human to find evidence of that link.

We know that motor neuron death in ALS is the culmination of multiple aberrant biological process involving also nonneuronal cells such microglia and astrocyte, what emerge from our data is that lipid homeostasis, oxidative stress and cellular remodelling are strictly related to ALS. We have just previously commented the role of the specific here identified variants in the cellular/molecular pathways. A recent finding has been reported on how lipid molecules can induce the cytotoxic aggregation of Cu/Zn superoxide dismutase, the major gene linked to the familial and sporadic form of the disease, under physiological conditions suggesting that it might provide a possible mechanism for the pathogenesis of ALS [28]. Recently, lower serum lipid levels are shown to be related to respiratory impairment in patients with ALS [29].In addition, in amyotrophic lateral sclerosis-parkinsonism dementia complex (ALS-PDC) common in the western Pacific area and repeatedly linked to the use of seeds of various species of cycad, it has been demonstrated in vitro the effects of cholesterol *β*-D-glucoside, cholesterol and cycad phytosterol glucosides on respiration and reactive oxygen species generation in brain mitochondria [30]. Indeed cholesterol homeostasis dysfunctions may lead to human brain disease such as Alzheimer's disease [31] and Huntington's disease [32], for example.

In a first work about ALS [6], we showed an evolutionary method to select the most predictive variables able to distinguish between ALS patients and controls. In that work the question was which are the independent variables whose a priori probability distribution separates in a better way cases from controls? A set of seven variables showed to do this job in a suitable manner (an average accuracy in blind testing of 96%).

In this work we pose to the scientific community a different question: which genetic polymorphisms (variables) protect or make more vulnerable the ALS patients and the control subjects?

There is not a necessary intersection between these two questions: small differences in an organ at work can produce big differences in symptoms, because of the interactions with other organs. Therefore, some polymorphisms can work as more evident symptoms of a disease without to be the main reason of that disease. In the same way, the seven variables of the previous work can be optimal predictors of the ALS, without to be the main reason of the ALS syndromes: they are useful to recognize the ALS, but they are not a necessary explanation of the ALS.

The more predictive features in a disease are not necessary the same features able to explain better the dynamics of that disease; an example: in the case of alcohol addiction, the main reason to become an alcoholic could be a sociopsychological condition, but the more predictive features to understand if someone is an alcoholic can be the analysis of the functional state of his/her liver.

In the actual work, using a completely new adaptive algorithm, we have tried to understand which genetic polymorphisms explain better the deep difference between Cases and Controls. In other words how all the polymorphisms are arranged in different networks, with different links and connections strength, into the two subsamples.

## 5. Conclusion

We applied here a revolutionary methodology able to deal with complex disease such as sporadic ALS. This new approach allowed to identify genes/SNPs conferring susceptibility or protection to the disease, we were not able to discriminate which allele of the six variants identified is really involved, and this is due to how the database was realized. From the dataset here analyzed we extrapolate biological information coherent with possible pathogenetic pathways related to ALS. Our data clearly demonstrate the power of this new approach and it would be of great interest to test with other more complex ALS database to get more information.

## Figures and Tables

**Algorithm 1:**
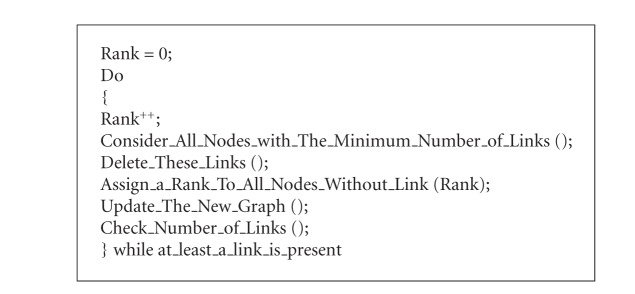
Pruning algorithm.

**Table 1:** The Delta *H* function with the relative hubness into the Controls and Cases datasets.

Control			
Variables	Hub relevance	Variables	Hub relevance
Global	0.17193	NOS3_C_690_T	0.171429
ADRB3_trp64arg	0.136905	NOS3_glu298asp	0.171429
LIPC_C_480_T	0.136905	DCP1_ins_del	0.171429
MMP3_5A_6A	0.136905	AGTR1_A1166C	0.171429
APOC3_C_641_A	0.171429	AGT_met235thr	0.171429
APOC3_C_482_T	0.171429	NPPA_G664A	0.171429
APOC3_T_455_C	0.171429	NPPA_T2238C	0.171429
APOC3_C1100T	0.171429	ADD1_gly460trp	0.171429
APOC3_C3175G	0.171429	SCNN1_trp493arg	0.171429
APOC3_T3206G	0.171429	SCNN1A_ala663thr	0.171429
APOE_cys112arg	0.171429	GNB3_C825T	0.171429
APOE_arg158cys	0.171429	ADRB2_arg16gly	0.171429
APOA4_thr347ser	0.171429	ADRB2_gln27glu	0.171429
PPARG_pro12ala	0.171429	APOB_thr71ile	0.171429
APOA4_glu360his	0.171429	F2_G20210A	0.171429
LPL_T_93_G	0.171429	F5_arg506gln	0.171429
LPL_asp9asn	0.171429	F7_del_ins	0.171429
LPL_asn291ser	0.171429	F7_arg353glu	0.171429
LPL_ser447term	0.171429	PAI_G5_G4	0.171429
PON1_met55leu	0.171429	PAI_G11053T	0.171429
PON1_gln192arg	0.171429	FGB_G_455_A	0.171429
PON2_ser311cys	0.171429	ITGA2_G873A	0.171429
LDLR_Ncol_Ncol	0.171429	ITGB3_leu33pro	0.171429
CETP_630	0.171429	SELE_ser128arg	0.171429
CETP_628	0.171429	SELE_leu554phe	0.171429
CETP_ile405val	0.171429	ICAM_gly214arg	0.171429
LTA_thr26asn_A	0.171429	TNFa_G_376_A	0.171429
MTHFR_C677T	0.171429	TNFa_G_308_A	0.171429
NOS3_A_922_G	0.171429	TNFa_244	0.171429
		TNFa_238	0.171429
		LTA_thr26asn_B	0.171429

	Cases		
Variables	Hub relevance	Variables	Hub relevance

Global	0.137127	AGTR1_A1166C	0.136905
APOA4_thr347ser	0.136905	AGT_met235thr	0.136905
APOB_thr71ile	0.136905	NPPA_G664A	0.136905
APOC3_C_641_A	0.136905	NPPA_T2238C	0.136905
APOC3_C_482_T	0.136905	ADD1_gly460trp	0.136905
APOC3_T_455_C	0.136905	SCNN1_trp493arg	0.136905
APOC3_C1100T	0.136905	SCNN1A_ala663thr	0.136905
APOC3_C3175G	0.136905	GNB3_C825T	0.136905
APOC3_T3206G	0.136905	ADRB2_arg16gly	0.136905
APOE_cys112arg	0.136905	ADRB2_gln27glu	0.136905
APOE_arg158cys	0.136905	MMP3_5A_6A	0.136905
ADRB3_trp64arg	0.136905	F2_G20210A	0.136905
PPARG_pro12ala	0.136905	F5_arg506gln	0.136905
LIPC_C_480_T	0.136905	F7_del_ins	0.136905
LPL_T_93_G	0.136905	F7_arg353glu	0.136905
LPL_asp9asn	0.136905	PAI_G5_G4	0.136905
LPL_asn291ser	0.136905	PAI_G11053T	0.136905
PON1_met55leu	0.136905	FGB_G_455_A	0.136905
PON1_gln192arg	0.136905	ITGA2_G873A	0.136905
PON2_ser311cys	0.136905	ITGB3_leu33pro	0.136905
LDLR_NcoI_NcoI	0.136905	SELE_ser128arg	0.136905
CETP_630	0.136905	SELE_leu554phe	0.136905
CETP_628	0.136905	ICAM_gly214arg	0.136905
CETP_ile405val	0.136905	TNFa_G_376_A	0.136905
LTA_thr26asn_A	0.136905	TNFa_G_308_A	0.136905
MTHFR_C677T	0.136905	TNFa_244	0.136905
NOS3_C_690_T	0.136905	TNFa_238	0.136905
NOS3_glu298asp	0.136905	LTA_thr26asn_B	0.136905
DCP1_ins_del	0.136905	APOA4_glu360his	0.171429
		NOS3_A_922_G	0.171429
		LPL_ser447term	0.171429
